# Supplementary material for: A magneto-activated nanoscale cytometry platform for molecular profiling of small extracellular vesicles
Source: Nat Commun. 2023 Sep 11;14:5576. doi: 10.1038/s41467-023-41285-8 (PMC10495366; doi:10.1038/s41467-023-41285-8)
Supplement: Supplementary file 1 — Supplementary Information [file 41467_2023_41285_MOESM1_ESM.pdf]

Supplementary Information

A Magneto-Activated Nanoscale Cytometry Platform for Molecular Profiling of Small Extracellular Vesicles

Kangfu Chen[1,9], Bill T.V. Duong[2,9], Sharif U. Ahmed[1], Piriththiv Dhavarasa[3], Zongjie Wang[7], Mahmoud Labib[1,5,6], Connor Flynn[2,5], Jingya Xu[2], Yi Y. Zhang[1], Hansen Wang[1], Xiaolong Yang[1], Jagotamoy Das[5], Hossein Zargartalebi[1], Yuan Ma[1], Shana O. Kelley[1,2,3,4,5,7,8]*

[1]Department of Pharmaceutical Sciences, University of Toronto, Toronto, Ontario, Canada

[2]Department of Chemistry, University of Toronto, Toronto, Ontario, Canada

[3]Department of Biochemistry, University of Toronto, Toronto, Ontario, Canada

[4]Institute for Biomaterials and Biomedical Engineering, University of Toronto, Toronto, Ontario, Canada

[5]Department of Chemistry, Northwestern University, Evanston, IL, USA.

[6]Peninsula Medical School, Faculty of Health, University of Plymouth, Plymouth, United Kingdom.

[7]Department of Biomedical Engineering, Northwestern University, Evanston, IL, USA

[8]Chan Zuckerberg Biohub Chicago, Chicago, IL, USA

[9]These authors contributed equally: Kangfu Chen, Bill Duong

*Corresponding author: shana.kelley@northwestern.edu

Contents

Supplementary Note 1. Optimization of NanoEPIC platform

Supplementary Note 2. ExoPD-L1 profiling of spiked sample using NanoEPIC

Supplementary Note 3. Calculation of the NanoEPIC score

Supplementary table index

Supplementary Table 1. Parameters for simulation

Supplementary Table 2. List of antibodies for western blot and flow cytometry

Supplementary Table 3. List of ELISA kits

Supplementary Table 4. Demographics of cancer patients

Supplementary figures index

Supplementary Figure 1. Design of the NanoEPIC device

Supplementary Figure 2. Overview of the NanoEPIC setup

Supplementary Figure 3. Cellular and exosomal PD-L1 expression in NSCLC cell lines

Supplementary Figure 4. TEM images of MNPs bound to sEVs

Supplementary Figure 5. Simulation of magnetic deflection within the NanoEPIC device

Supplementary Figure 6. Assessment of the specificity of the NanoEPIC platform

Supplementary Figure 7. Simulations of sample flow rate in the NanoEPIC device

Supplementary Figure 8. Assessment of the performance of NanoEPIC device using sEVs from different NSCLC cell lines and in human plasma

Supplementary Figure 9. Evaluation of MNP release from sEVs

Supplementary Figure 10. In vivo PD-1 immunotherapeutic model

Supplementary Figure 11. exoPD-L1 profiling of PD-1 immunotherapeutic mouse model

Supplementary Figure 12. Assessment of the correlation between cPD-L1 and exoPD-L1

Supplementary Figure 13. Flow cytometric analysis of TILs from a murine immunotherapeutic model

Supplementary Figure 14. Imaging of sEVs bound to T cells

Supplementary Figure 15. Flow cytometric analysis of granzyme B, ki67, and CD69 from T cells after treatment with sEVs

Supplementary Figure 16. Illustration of microfabrication steps for the NanoEPIC device

Supplementary Figure 17 NanoEPIC score of patients before and after anti-PD1/anti-PD-L1 immunotherapy.

Supplementary Note 1. Optimization of NanoEPIC platform. NanoEPIC relies on magnetic deflection to sort sEV subpopulations. To interrogate the motion of an sEV, we first simulated the flow field and the magnetic field in the flow microchannel using COMSOL Multiphysics. Supplementary Fig. 1a shows the side-view layout of the NanoEPIC system for simulation in two dimensions. It consists of a permanent magnet, a glass substrate with magnetic guides, and a flow microchannel. Supplementary Fig. 1b shows the magnetization of a magnetic guide in the presence of permanent magnets along with the magnetic field inside the microchannel while under the influence of the magnetic guide. The magnetic field is highest near the edges of a magnetic guide upon magnetization. As a result, the abrupt change in the magnetic field induces a strong magnetic gradient (Fig. 2c) at the edges of the guides. The magnetic gradient is proportional to the magnetic force exerted on the sEV, which will be discussed in detail later.

Next, the flow field is simulated in the microchannel. The mixing of sample flow and buffer flow near the inlet along with the separation of the bulk flow were the main concerns for the design optimizations. The goal is to focus the sample flow to the negative outlet in the absence of an external magnetic field. For the NanoEPIC system, a withdraw pumping is used for sample processing. We set the ratio of flow rates between the negative, low, medium, and high outlets as 3:1:1:1 respectively. The flow resistance of the sample outlet is adjusted to be slightly higher than that of the buffer outlet (11:10) so that the ratio of buffer flow is slightly higher than the ratio of sample flow. As shown in Supplementary Fig. 2a and 2b, the sample flow is precisely focused to the negative outlet.

To simulate the magnetic deflection of an sEV, we have developed a theoretical model to describe the motion of a magnetically labeled sEV. The sEV is modeled as a rigid sphere with a diameter of 100 nm. As shown in Supplementary Fig. 1d, the sEV is subjected to the magnetic force and the Stokes' drag force in the microchannel. The magnetic force $\boldsymbol{F}_m$ can be expressed as:[53]

$$\boldsymbol{F}_m = N_{bead} V_{bead} (\boldsymbol{M} \cdot \boldsymbol{\nabla}) \boldsymbol{B} \quad (1)$$

Where N_{bead} is the number of magnetic nanoparticles (MNPs) bonded to the sEV; V_{bead} is the volume of MNP; $\boldsymbol{M}$ net magnetic moment of the MNPs attached; $\boldsymbol{B}$ the magnetic flux density.

The Stokes' drag force can be expressed as

$$\boldsymbol{F}_d = 6\pi\eta r_p \Delta\boldsymbol{U} \quad (2)$$

Where η is the viscosity of the fluid medium; r_p is the radius of the sEV; $\Delta\boldsymbol{U}$ is the relative velocity between fluid flow and the sEV.

The kinetic motion of the sEV is described by the dynamic equation where the magnetic force and drag force are the main determinants of the acceleration. A local coordinate system is set as shown in Supplementary Fig. 1d, where the x-axis points to the direction normal to the magnetic guide, and the y-axis is parallel to the channel height. Eq. 3 and Eq. 4 are given to describe the motion of the sEV across the magnetic guides and along the channel height. The motion of the sEV along the channel height shows whether the sEV can be brought to the bottom of the microchannel where the magnetic gradient reaches the maximum (Supplementary Fig. 1c). The motion of the sEV across the magnetic guide ultimately determines if the sEV can be deflected by the magnetic guide.

$$m_p \frac{\mathrm{d}U_{px}}{\mathrm{d}t} = 6\pi\eta r_p(U_f sin\theta - U_{px}) - F_{mx} \quad (3)$$

$$m_p \frac{\mathrm{d}U_{py}}{\mathrm{d}t} = -6\pi\eta r_p U_{py} + F_{my} \quad (4)$$

Where m_p is the mass of the sEV; U_{px} is the velocity of the sEV along the x-direction perpendicular to the magnetic guide; η is the viscosity of the sample solution; r_p is the radius of the sEV; θ is the deflection angle of the magnetic guide; U_{fx} is the x-component of flow velocity ($U_{fx} = U_f sin\theta$); F_m is the magnetic force the sEV experiences.

At a time-interval Δt, U_{fx} and F_m are considered constants. U_{px} can then be expressed as

$$U_{px} = U_{fx} - \frac{F_{mx}}{6\pi\eta r_p}(1 - e^{-\frac{6\pi\eta r_p}{m_p}\Delta t}) \quad (5)$$

$$U_{py} = \frac{F_{my}}{6\pi\eta r_p}(1 - e^{-\frac{6\pi\eta r_p}{m_p}\Delta t}) \quad (6)$$

Since m_p is extremely small, $e^{-\frac{6\pi\eta r_p}{m_p}\Delta t}$ is close to zero. Therefore, U_{px} can be simplified as

$$U_{px} = U_{fx} - \frac{F_{mx}}{6\pi\eta r_p} \quad (7)$$

$$U_{py} = \frac{F_{my}}{6\pi\eta r_p} \quad (8)$$

If the sEV follows the magnetic guide and is deflected, it requires

$$U_{px} = 0 \quad (9)$$

Therefore,

$$F_{mx} = 6\pi\eta r_p\ U_f sin\theta \quad (10)$$

$$N_{bead} V_{bead} (\boldsymbol{M} \cdot \boldsymbol{\nabla}) B_x = 6\pi\eta r_p\ U_f sin\theta \quad (11)$$

For a given flow rate where U_f is constant, the number of MNPs (N_{bead}) required to achieve magnetic deflection of an sEV is proportional to the deflection angle (θ). Since the number of MNPs attached to the sEV is proportional to the expression of target proteins, we can perform protein expression-based profiling by designing different deflection angles.

Since the magnetic gradient decreases dramatically from the bottom to the top of the microchannel (Supplementary Fig. 1c), bringing the sEV down to the bottom of the microchannel is necessary for magnetic deflection (Supplementary Fig. 2d). In this simulation, the worst-case scenario is considered where the sEV was released from the top of the microchannel. The flow path of the sEV along the channel height was predicted. To describe the motion of the sEV in the x-y frame, we derived the differential equations in both the x-direction and y-direction, as given in Eq. 12 and Eq. 13.

$$dX = U_{px} dt = \left(U_{fx} - \frac{F_{mx}}{6\pi\eta r_p}\right) dt \quad (12)$$

$$dY = U_{py}dt = \frac{F_{my}}{6\pi\eta r_p}dt \tag{13}$$

Where

$$F_{mx} = N_{bead}V_{bead}(\boldsymbol{M}\cdot\boldsymbol{\nabla})B_x(X,Y) \tag{14}$$

$$F_{my} = N_{bead}V_{bead}(\boldsymbol{M}\cdot\boldsymbol{\nabla})B_y(X,Y) \tag{15}$$

The position of the sEV based on accumulated time can be expressed as Eq. 16 and Eq. 17.

$$X_i = X_{i-1} + dX_{i-1} = X_{i-1} + \left(U_{fx_{i-1}} - \frac{F_{mx_{i-1}}}{6\pi\eta r_p}\right)dt \tag{16}$$

$$Y_i = Y_{i-1} + dY_{i-1} = Y_{i-1} + \frac{F_{my_{i-1}}}{6\pi\eta r_p}dt \tag{17}$$

Where

$$F_{mx_{i-1}} = N_{bead}V_{bead}(\boldsymbol{M}\cdot\boldsymbol{\nabla})B_x(X_{i-1},Y_{i-1}) \tag{18}$$

$$F_{my_{i-1}} = N_{bead}V_{bead}(\boldsymbol{M}\cdot\boldsymbol{\nabla})B_y(X_{i-1},Y_{i-1}) \tag{19}$$

With Eq. 16 and Eq. 17, we were able to sketch the flow path of the sEV in the microchannel using MATLAB (R2020a). If the sEV passes all the magnetic guides ($X_i >$ position of the last magnetic guide), it will be considered undeflected. For other conditions, the sEV is brought to the bottom of the channel and its deflection depends on the difference between the magnetic force and the drag force as shown in Eq 10. The

Supplementary Note 2. ExoPD-L1 profiling of spiked sample using NanoEPIC. To determine whether NanoEPIC can be used to perform exoPD-L1 profiling on circulating sEVs in clinical samples, we spiked H1975 sEVs in plasma samples collected from healthy donors and processed the anti-PD-L1 MNP-treated spiked samples with NanoEPIC. Compared with control plasma samples, the deflection efficiency for the spiked samples was significantly higher (Supplementary Fig. 8d). For the distribution pattern, the percentage of exo-M and exo-H sEV subpopulations for the spiked samples are significantly higher compared with the control samples (Supplementary Fig. 8e). These results demonstrate the feasibility of NanoEPIC for exoPD-L1 profiling of circulating sEVs in blood plasma.

Supplementary Note 3. Calculation of the NanoEPIC score. The NanoEPIC platform has the capacity to sort sEVs based on PD-L1 expression at high throughput and high resolution and with this reasoning, it can also be utilized for exosomal profiling. While the distribution profiles from each sorting round allow for a visual comparison between samples, generating a single numerical score that can summarize these profiles can grant the ability to perform quantitative analysis and allow for better management of larger sample sizes in clinical studies.

Based on the design of the NanoEPIC assay, there are mainly two considerations in generating the NanoEPIC score: i) deflection efficiency (DE), and ii) profile distribution. The DE is defined as the number of total deflected sEVs (including the sEVs collected from L, M, H outlets) over the number of total sEVs introduced to the NanoEPIC system. The profile distribution is defined as the ratios of sEVs collected from a certain outlet (L, M, or H) over the total deflected sEVs. Typically, samples with a larger proportion of sEVs deflected towards the high outlet suggest an

overall higher expression of PD-L1 per sEV. We can therefore infer that the distribution profile of deflected sEVs can represent the weighted average expression of PD-L1 per sEV. On the other hand, deflected sEVs only consist of a subset of the whole population of sEVs. Thus, we also need to consider the DE which corresponds to the proportion of PD-L1 positive sEVs in a sample. Based on these premises, we can simply define our NanoEPIC score as such:

$$NanoEPIC\ score = DE \times weighted\ average\ PDL1\ expression\ of\ deflected\ exosomes \tag{20}$$

Since the NanoEPIC platform operates through immunomagnetic sorting, we used Bead Average (BA) (i.e., the average number of anti-PD-L1 conjugated MNPs attached to sEVs) to approximate the relative PD-L1 expression per sEV. Simply, BA is defined as the weighted average of anti-PD-L1 conjugated MNPs per sEV. The NanoEPIC score is thus represented as follows:

$$NanoEPIC\ score = DE \times BA \tag{21}$$

DE can be further extracted from the following expression:

$$DE = \frac{Deflected\ exosomes}{Total\ exosomes} \times 100 = \frac{100 \cdot \sum_{i=1}^{3} E_i}{\sum_{i=1}^{4} E_i} \tag{22}$$

Where E_i represents total sEVs collected from outlet i and the assignment of i for each outlet is as follows: 1 = high, 2 = medium, 3 = low, 4 = negative.

To determine the BA, we first introduced a new term, σ_i^*, which represents the relative number of MNPs bound to sEVs from outlet i compared to sEVs from the low outlet. To determine σ_i^*, we can refer to the projected magnitude of magnetic deflection which is proportional to the sine of the deflection angles as given in Eq. 10. As such, the σ_i^* values are as follow:

$$\sigma_1^* = \sin 10°/\sin 3° = 3.32 \tag{23}$$

$$\sigma_2^* = \sin 5°/\sin 3° = 1.67 \tag{24}$$

$$\sigma_3^* = \sin 3°/\sin 3° = 1.00 \tag{25}$$

We can improve our estimate of BA from experimental data from TEM imaging (Fig. 2d and Supplementary Fig. 4). From our analysis, we can impend a correction value of 4 onto σ_i^* to better approximate the BA values from each outlet. Therefore, our corrected σ_i^* values (σ_i) are as follows:

$$\sigma_1 = 4\sigma_1^* = 13.27 \tag{26}$$

$$\sigma_2 = 4\sigma_2^* = 6.66 \tag{27}$$

$$\sigma_3 = 4\sigma_3^* = 4.00 \tag{28}$$

To calculate the overall BA of a sample, we can use the following expression:

$$BA = \frac{\sum_{i=1}^{3}(\sigma_i \cdot E_i)}{\sum_{i=1}^{3} E_i} \tag{29}$$

Combining Eq. 22 and Eq. 29, the NanoEPIC score can be calculated as such:

$$NanoEPIC\ score = \frac{100 \cdot \sum_{i=1}^{3}(\sigma_i \cdot E_i)}{\sum_{i=1}^{4} E_i} \tag{30}$$

Supplementary Table 1. Parameters for simulation

Parameter	Value	Unit	Description
r_p	5	nm	Radius of the MNP
r	50	nm	Radius of an sEV
η	10^{-3}	Pa	Viscosity of sample flow
M	80	A·m^2/kg	Saturated magnetization of the MNP
ρ	5170	Kg/m^3	Density of the MNP

Supplementary Table 2. List of antibodies for western blot and flow cytometry

Marker	Application	Reactivity	Dilution	Catalog Number	Vendor
CD274	WB	Human	1:1000	13684	Cell Signaling Technology
CD274	WB	Mouse	1:500	14-5982-85	Thermofisher
CD63	WB	Human	1:1000	ab271286	Abcam
CD9	WB	Human	1:1000	ab236630	Abcam
Anti-rabbit, HRP	WB	Rabbit	1:10000	31460	Thermofisher
Anti-mouse, HRP	WB	Mouse	1:10000	31430	Thermofisher
Anti-mouse IgG, Alexa Fluor 488	FC	Mouse	1 μg/mL	A28175	Thermofisher
CD274, APC	FC	Human	1 μg/mL	17-5983-42	Thermofisher
Ki67, PE	FC	Human, Mouse, Rat, Rhesus	1:20	567719	BDbiosciences
Ki67, APC	FC	Dog, Human	1:20	17-5699-42	Thermofisher
TCF7, PE	FC	Mouse, Human	1:20	564217	BDbiosciences
CD69, PE	FC	Mouse	1:20	12-0691-83	Thermofisher
CD69, Super Bright 436	FC	Human	1:20	62-0699-42	Thermofisher
CD137, PE	FC	Mouse	1:20	558976	BDbiosciences
IFN-γ, PE	FC	Mouse	1:20	554412	BDbiosciences
Granzyme B, PE	FC	Mouse	1:20	12-8898-82	Thermofisher
Granzyme B (GB11), PE-Cyanine5.5	FC	Human	1:20	GRB18	Thermofisher
PD-1, PE	FC	Mouse	1:20	566831	BDbiosciences
CD152, PE	FC	Mouse	1:20	130-116-390	Miltenyi Biotec
CD8a, APC	FC	Mouse	1:20	561093	BDbiosciences
CD8a, Super Bright 600	FC	Human	1:20	63-0088-42	Thermofisher
CD45, eFluor 450	FC	Mouse	1:20	48-0451-82	Thermofisher

Supplementary Table 3. List of ELISA kits

Marker	Reactivity	Reference / Catalog Number	Vendor
PD-L1	Human	BMS2212	Thermofisher
PD-L1	Mouse	DY1019-05	R&D Systems
IL-2	Human	BMS221HS	Thermofisher
IFN-γ	Human	BMS228HS	Thermofisher

Supplementary Table 4. Demographics of cancer patients

Cancer Type	Suspected Clinical Stage at Blood Collection	Immune Checkpoint Inhibitor	Age at Baseline	Gender
Breast	T1aNXMX	Trastuzumab	50	Female
Lung	III	Pembrolizumab	47	Female
Lung	IIIA	Nivolumab	76	Female
Lung	III	Pembrolizumab	59	Female
Lung	N/A	Durvalumab	70	Female
Lung	IIIC	Nivolumab	74	Female
Endometrial	IV	Bevacizumab	48	Female
Bladder	N/A	Avelumab	73	Male
Colon	IV	Bevacizumab	41	Male
Kidney	N/A	Avelumab	67	Female

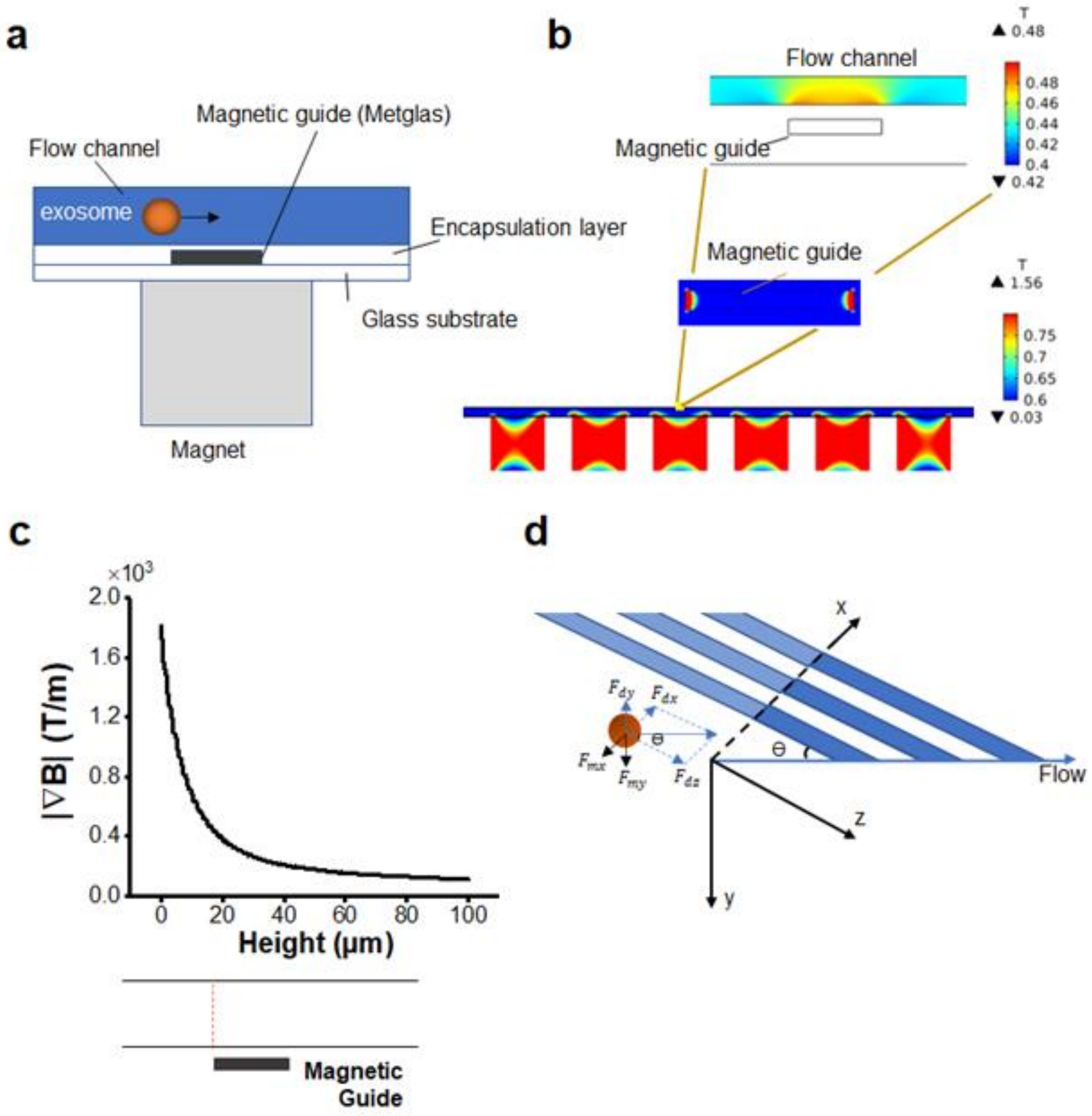

Supplementary Figure 1. Design of the NanoEPIC device. a, The layout of NanoEPIC for simulation. It consists of a permanent magnet, a glass substrate with magnetic guides, and a microfluidic channel. **b**, Simulation of the magnetic field in NanoEPIC with external magnets placed underneath. When the magnetic guide is magnetized, it governs the magnetic field in the microchannel. **c**, The magnetic gradient along the channel height at the edge of the magnetic guide as illustrated in the lower figure. The magnetic gradient decreases dramatically from the bottom to the top of the microchannel. **d**, The set local coordinate system in the microchannel to simplify the description of the kinetic motion of the sEV. X-direction points to the direction normal to the magnetic guide. Y-direction points to the bottom of the microchannel along the channel height. Z-direction is parallel to the magnetic guide. Source data are provided as a source data file.

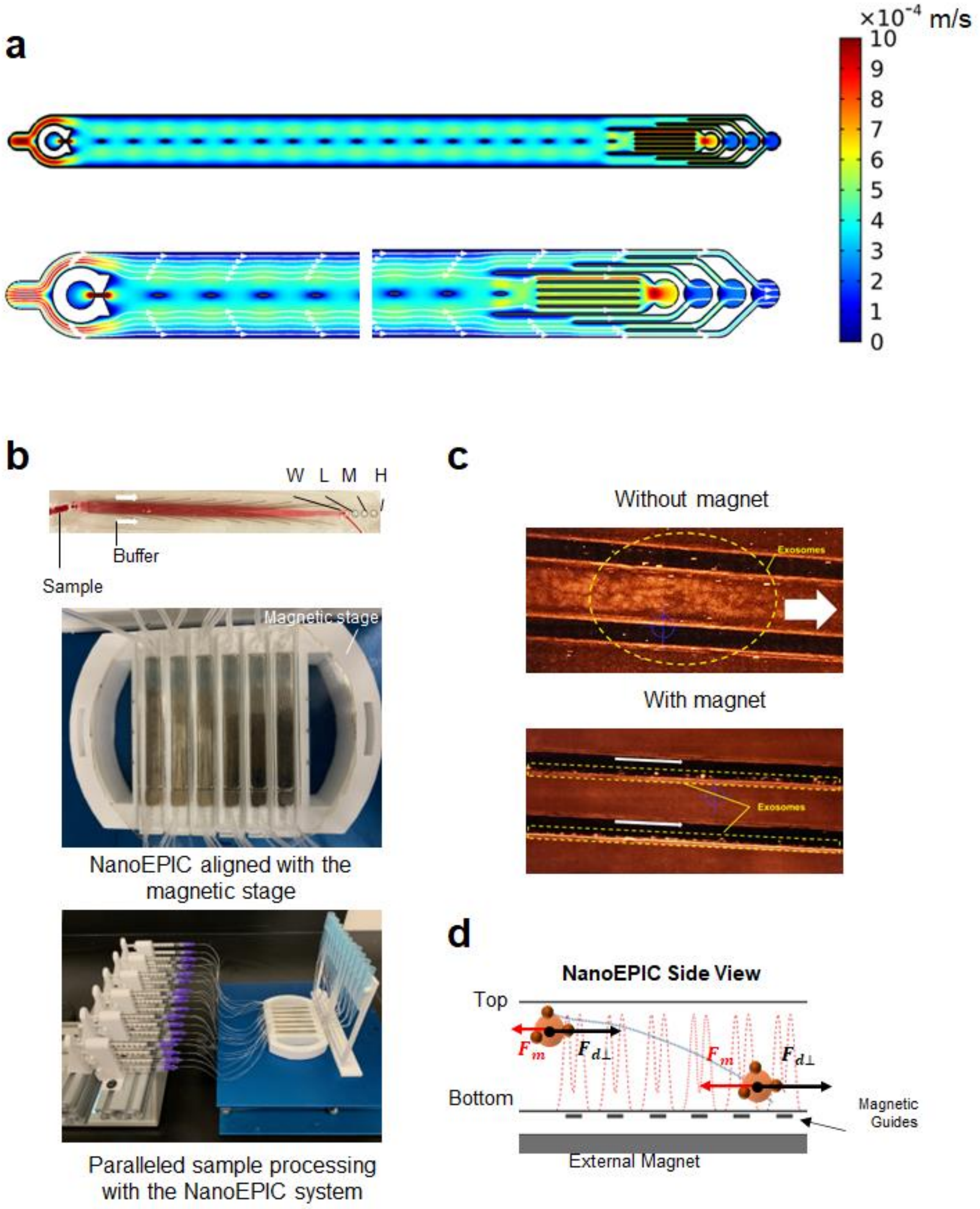

Supplementary Figure 2. Overview of the NanoEPIC setup. a, The simulation of the flow field in NanoEPIC. The sample flow is focused by the buffer flow and is collected in the negative outlet. **b**, Top figure shows the flow focusing of the sample, indicated by red food dye (sample) and clear buffer. The middle figure shows the NanoEPIC chips aligned with six permanent magnets mounted on a fixture. The NanoEPIC system includes 6 chips which are assembled on one glass wafer. It allows for the parallel processing of 6 different samples, allowing for high sample throughput. The bottom figure shows the parallel process of 6 samples using the NanoEPIC system. **c**, Top figure: the sEV sample in NanoEPIC without external magnetic field passes magnetic guides without deflection. Bottom figure: magnetically labeled sEVs follow the magnetic guide and are deflected when an external magnetic field is applied. **d**, Side view of the NanoEPIC device, illustrating the migration of magnetically labeled sEVs towards the bottom of the device during processing.

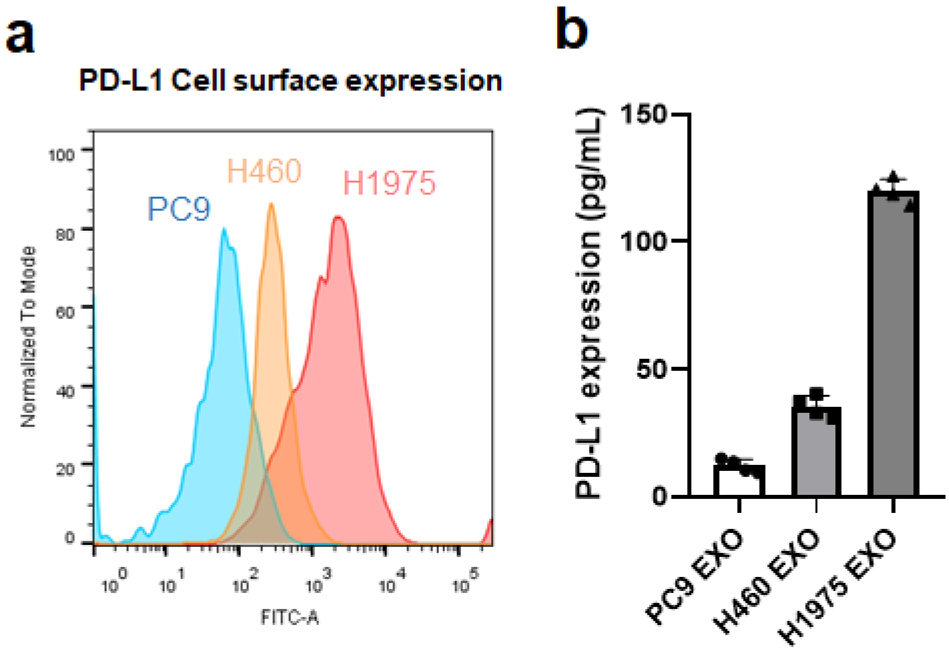

Supplementary Figure 3. Cellular and exosomal PD-L1 expression in NSCLC cell lines. a, cPD-L1 expression in PC9, H460, and H1975 cells. **b**, exoPD-L1 expression from the three cell lines measured by PD-L1 ELISA. (n=4, data represents mean ± s.d.) Source data are provided as a source data file.

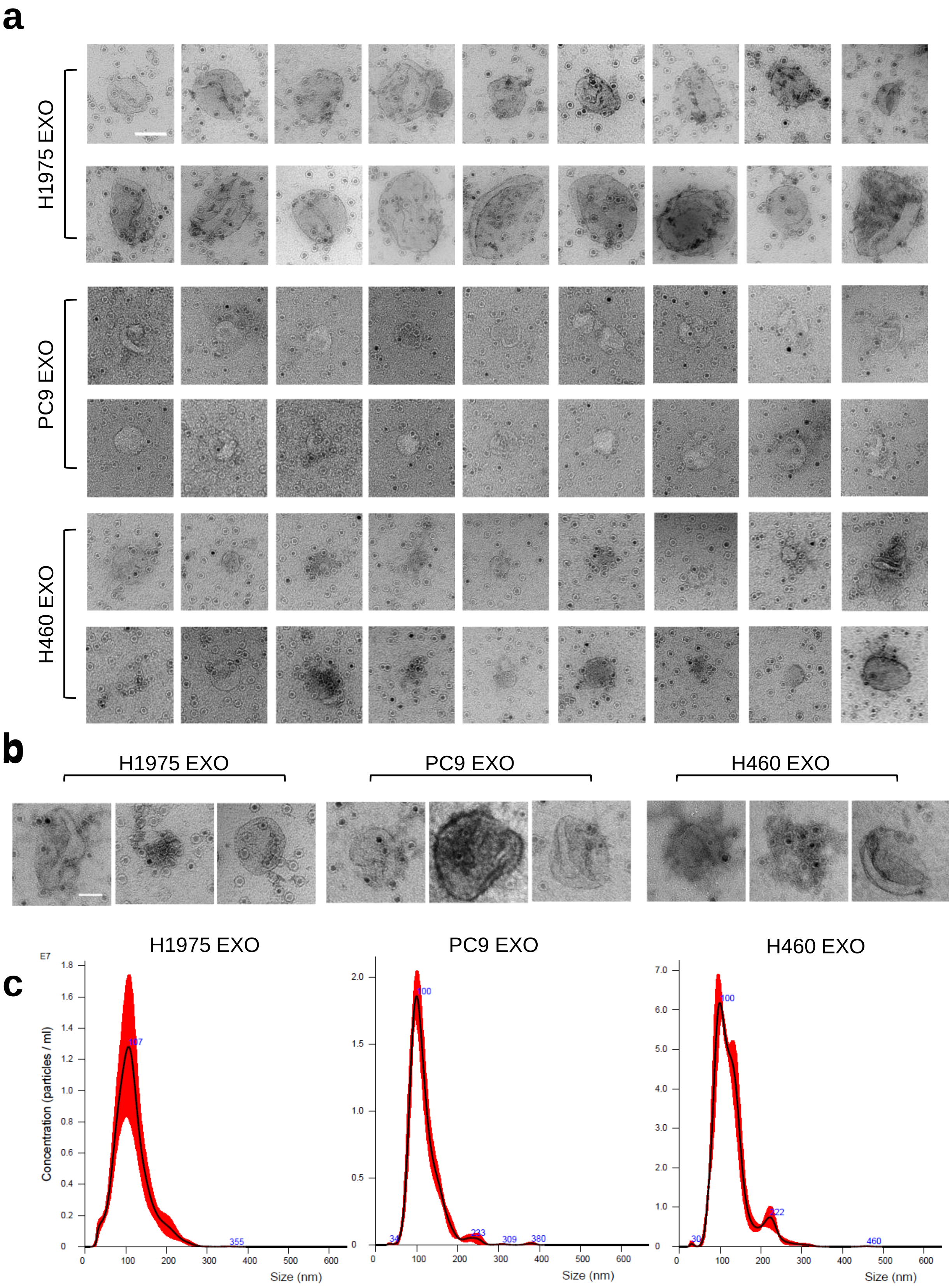

a
H1975 EXO
PC9 EXO
H460 EXO
b
H1975 EXO
PC9 EXO
H460 EXO
c
H1975 EXO
PC9 EXO
H460 EXO
E7
Concentration (particles / ml)
Size (nm)
107
355
34
100
223
309
380
30
100
222
460

Supplementary Figure 4. TEM images of MNPs bound to sEVs. a, Binding of sEVs with anti-PD-L1 conjugated MNPs. SEVs of different cell lines, including H1975 cells, PC9 cells, and H460 cells were used. The scale bar is 100 nm. **b**, MNP conjugated sEVs with bigger magnifications. The scale bar is 50 nm. **c**, NTA analysis of sEVs from H1975, PC9 and H460. The size distribution of the three types are similar to each other. H1975 EV has a mode diameter of 101.4 nm; PC9 EV has a mode diameter of 100.4 nm; H460 EV has a mod diameter of 100.8 nm. Source data are provided as a source data file.

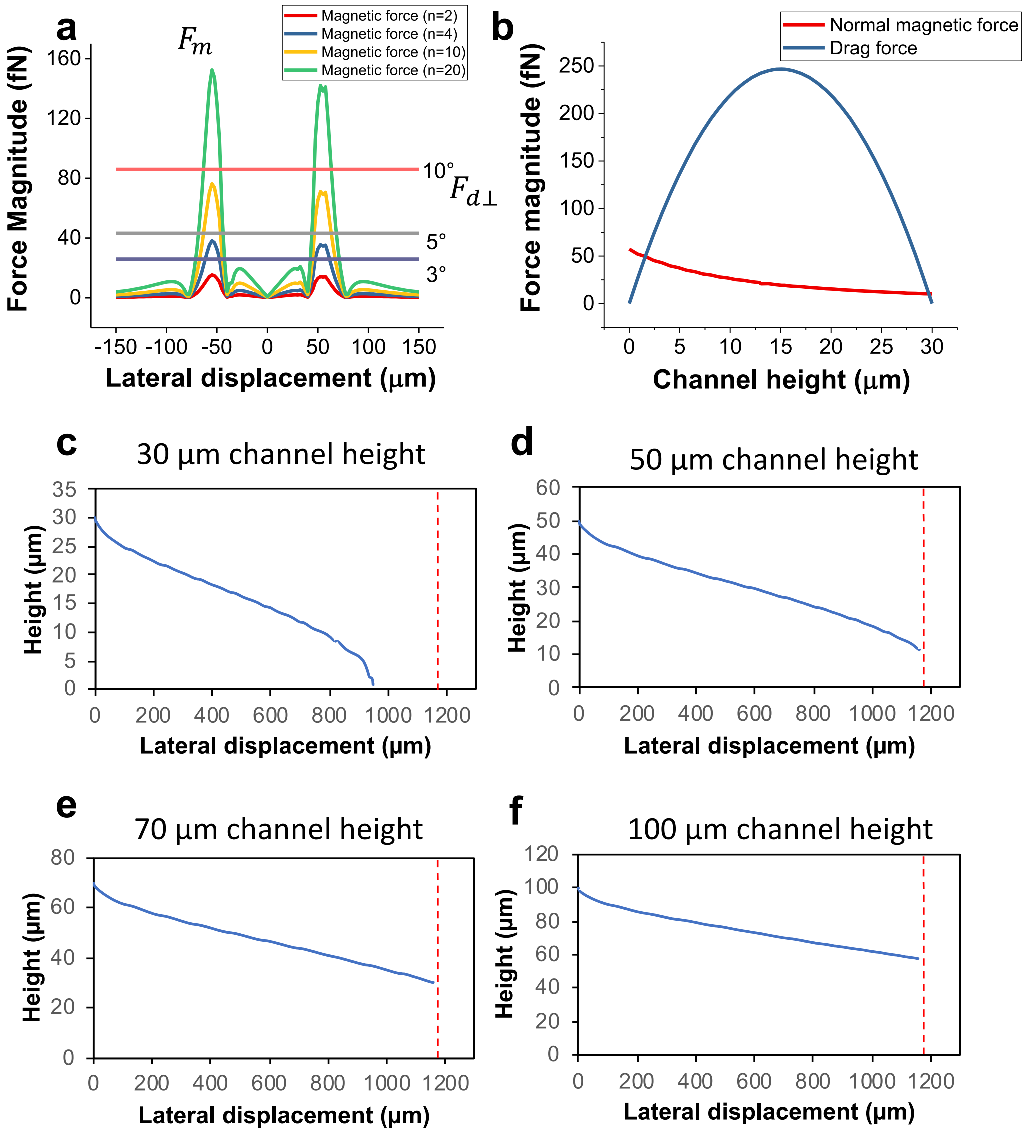

Supplementary Figure 5. **Simulation of magnetic deflection within the NanoEPIC device. a**, Magnetic force acting on an sEV with different numbers of MNPs (n = 2, 4, 10, 20) across the magnetic guide at the bottom of the channel compared with the drag forces in the direction of the magnetic guides. The magnetic guides have different deflection angles (3°, 5°, 10°). It illustrates the number of MNPs required to achieve magnetic deflection of the sEV at different deflection angles. **b**, Comparison between the drag force (assuming a deflection angle of 3°) and magnetic force (assuming 4 MNPs bound to the sEV) at the edge of a magnetic guide is simulated along the channel height. It requires the magnetic force to be higher than the drag force in the direction normal to the magnetic guide to achieve magnetic deflection. **c-f**, Numerical simulation of the flow path of an sEV with 4 bound MNPs starting from the top of the channel (30 -100 µm). The red line marks the position of the last magnetic guide. It shows that the sEV is brought to the bottom of the channel before passing the last magnetic guide. For **a-f**, the flow velocities are all set to be 1 mm/s, corresponding to a flow rate of 200 µL/h. Source data are provided as a source data file.

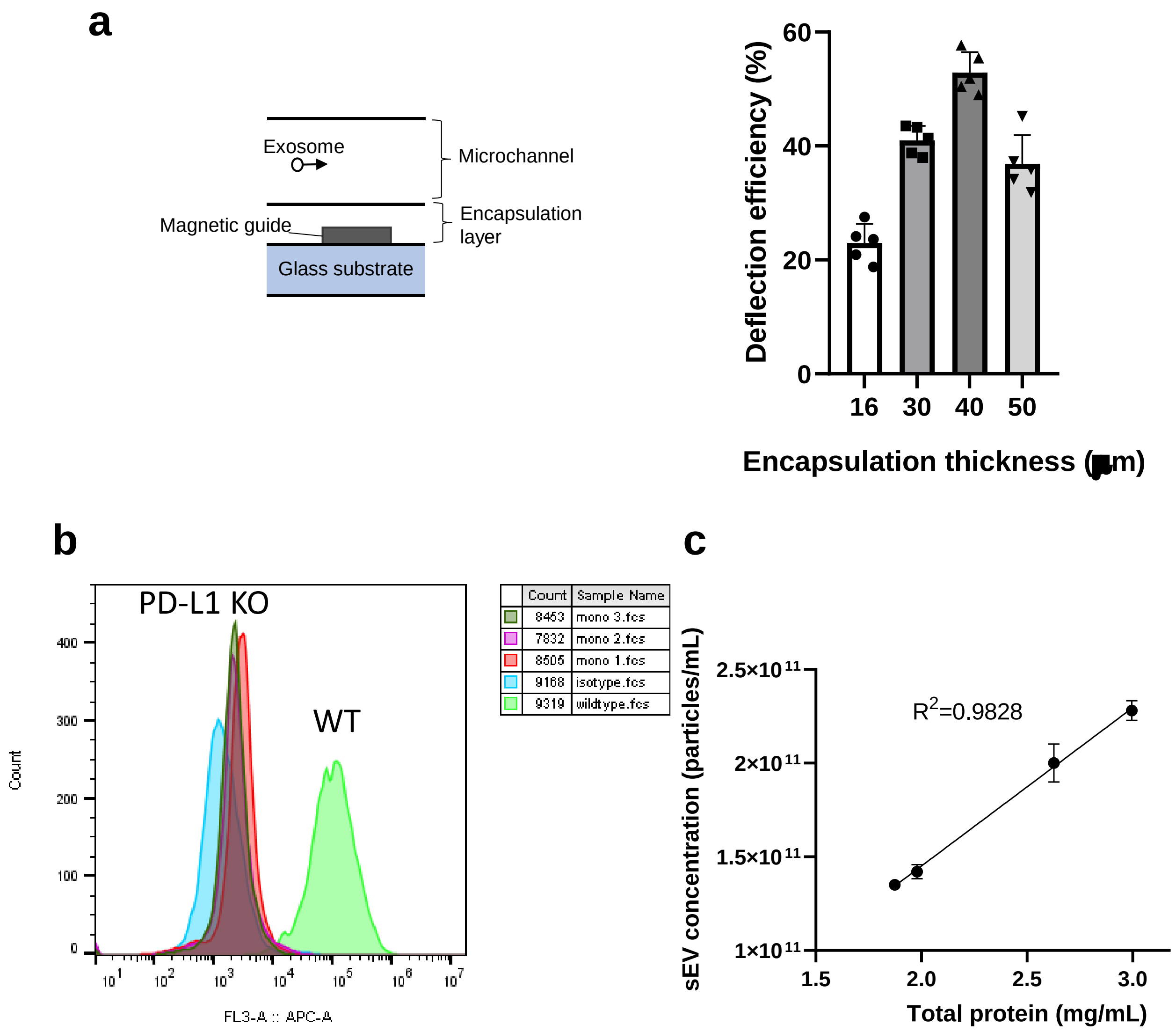

Supplementary Figure 6. Assessment of the specificity of the NanoEPIC platform. a, Deflection efficiency of H1975 sEVs in NanoEPIC with different encapsulation thicknesses. The encapsulation layer defines the separation between the magnetic guides and the flow microchannel. Small encapsulation layer results in significant trapping of sEVs in the device due to an increase in magnetic field. Larger encapsulation results in reduced deflection due to a weaker magnetic field experienced throughout the microchannel. (n=5, data represents mean ± s.d.) **b**, Analysis of PD-L1 in PD-L1 KO of H1975 cells using flow cytometry. **c**, Concentration of sEVs from H1975 vs the total protein of the sEVs. The total protein of sEVs shows good linearity with its concentration. Source data are provided as a source data file.

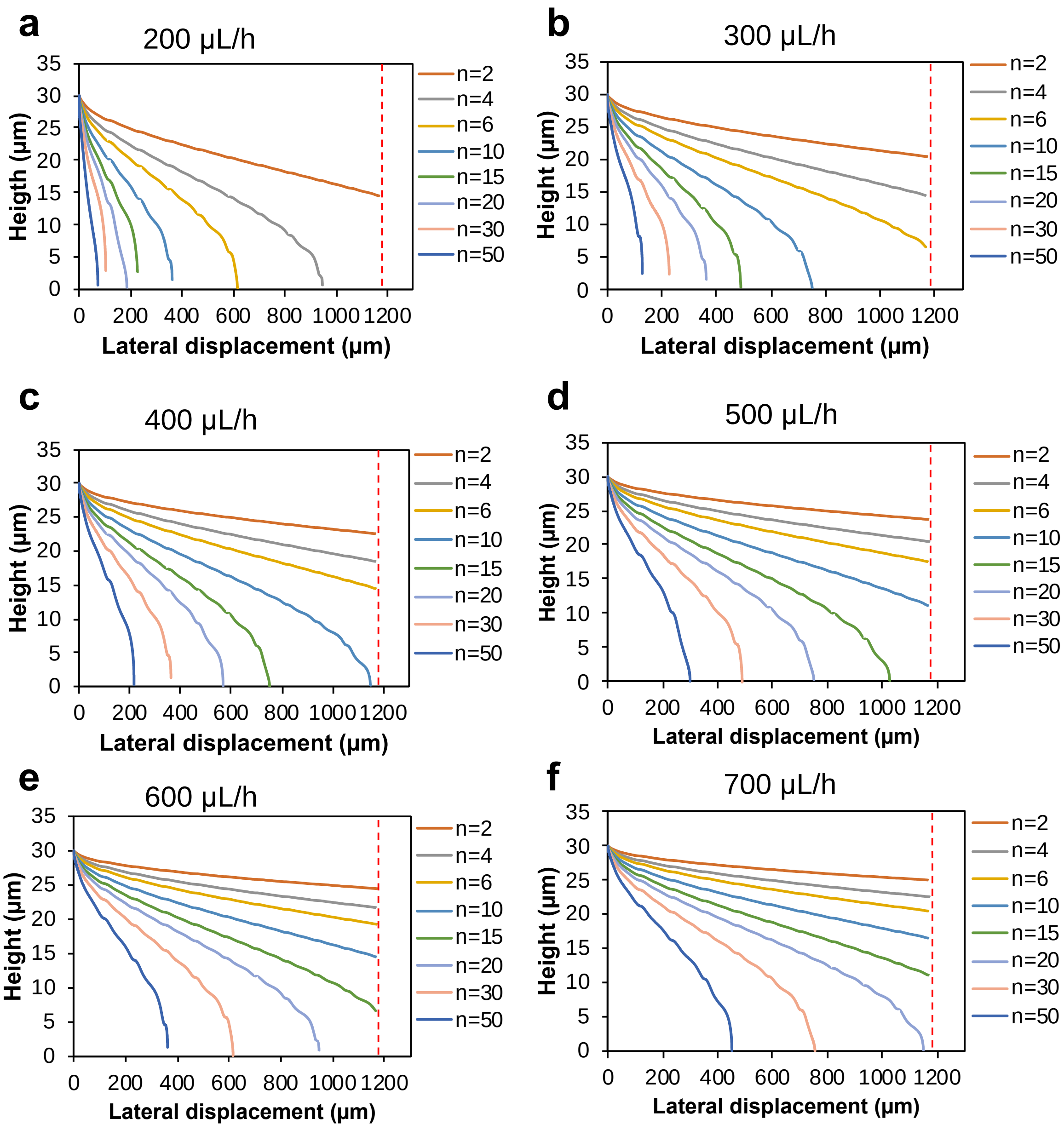

Supplementary Figure 7. Simulations of sample flow rate in the NanoEPIC device. Numerical simulation of flow paths of sEVs with different numbers of bound MNPs at different flow rates in the NanoEPIC device with an optimized channel height of 30 µm. These graphs illustrate the number of MNPs required to bring the sEV from the top to the bottom of the microchannel in order to fully experience magnetic deflection. The red lines mark the position of the final magnetic guide (closest to the outlet). If the flow path of the sEV intercepts with the red line, the sEV will not be deflected. Six different flow rates were tested, including 200 µL/h (**a**), 300 µL/h (**b**), 400 µL/h (**c**), 500 µL/h (**d**), 600 µL/h (**e**) and 700 µL/h (**f**). Source data are provided as a source data file.

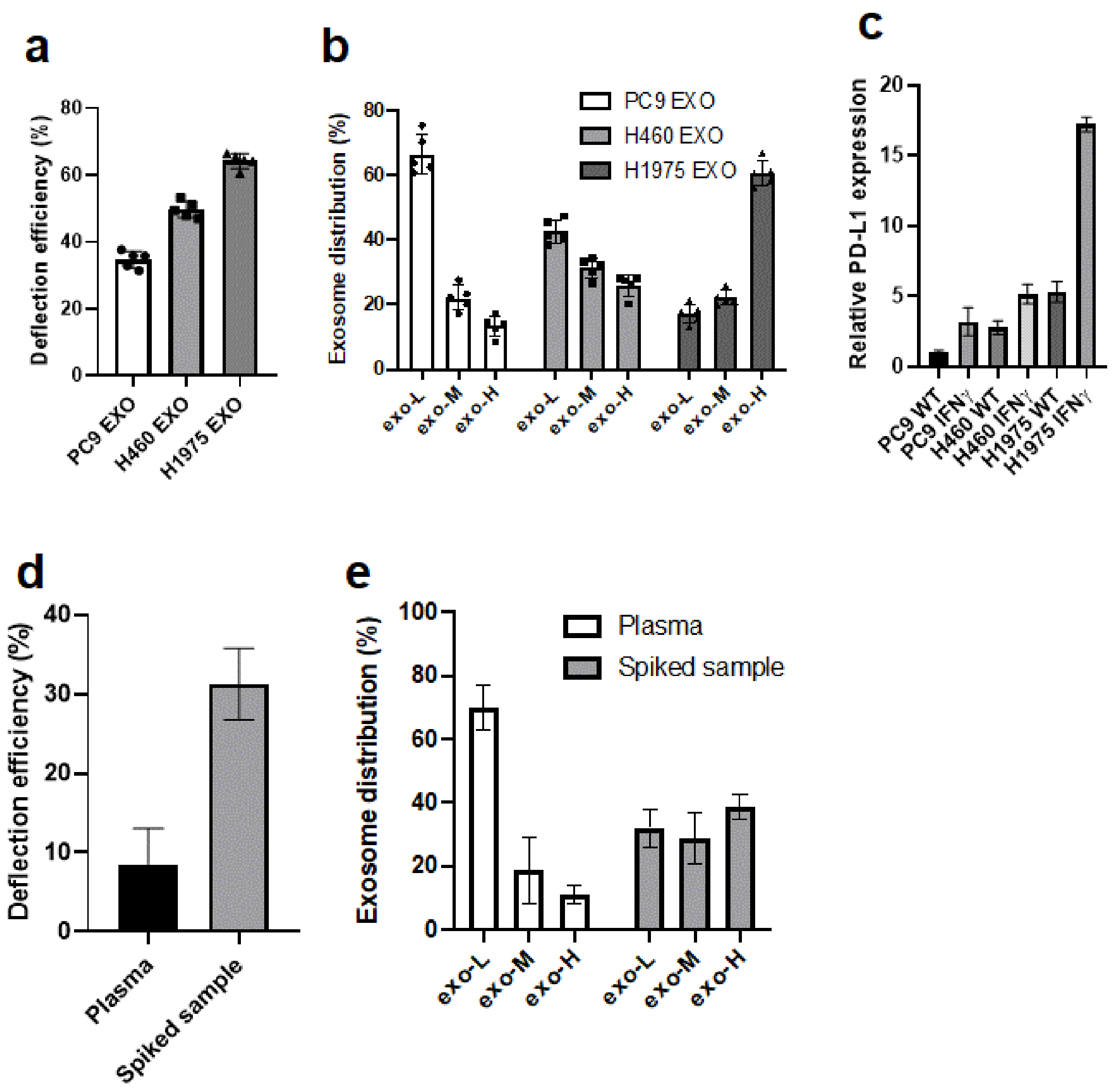

Supplementary Figure 8. **Assessment of the performance of NanoEPIC device using sEVs from different NSCLC cell lines and in human plasma. a**, Deflection efficiency of sEVs from different cell lines, including PC9, H460, and H1975. **b**, ExoPD-L1 profiling of different cell lines. **c**, Relative PD-L1 expression of unsorted sEVs collected from PC9, H460, and H1975 cells with/without IFN-γ treatment acquired through PD-L1 ELISA. PD-L1 levels were normalized to PC9 WT. SEVs from H1975 cells were spiked in human plasma and processed through the NanoEPIC device for PD-L1 sorting. Deflection efficiency (**d**) and exosomal distribution (**e**) demonstrate the high recovery and feasibility of exoPD-L1 profiling in clinical samples. Source data are provided as a source data file.

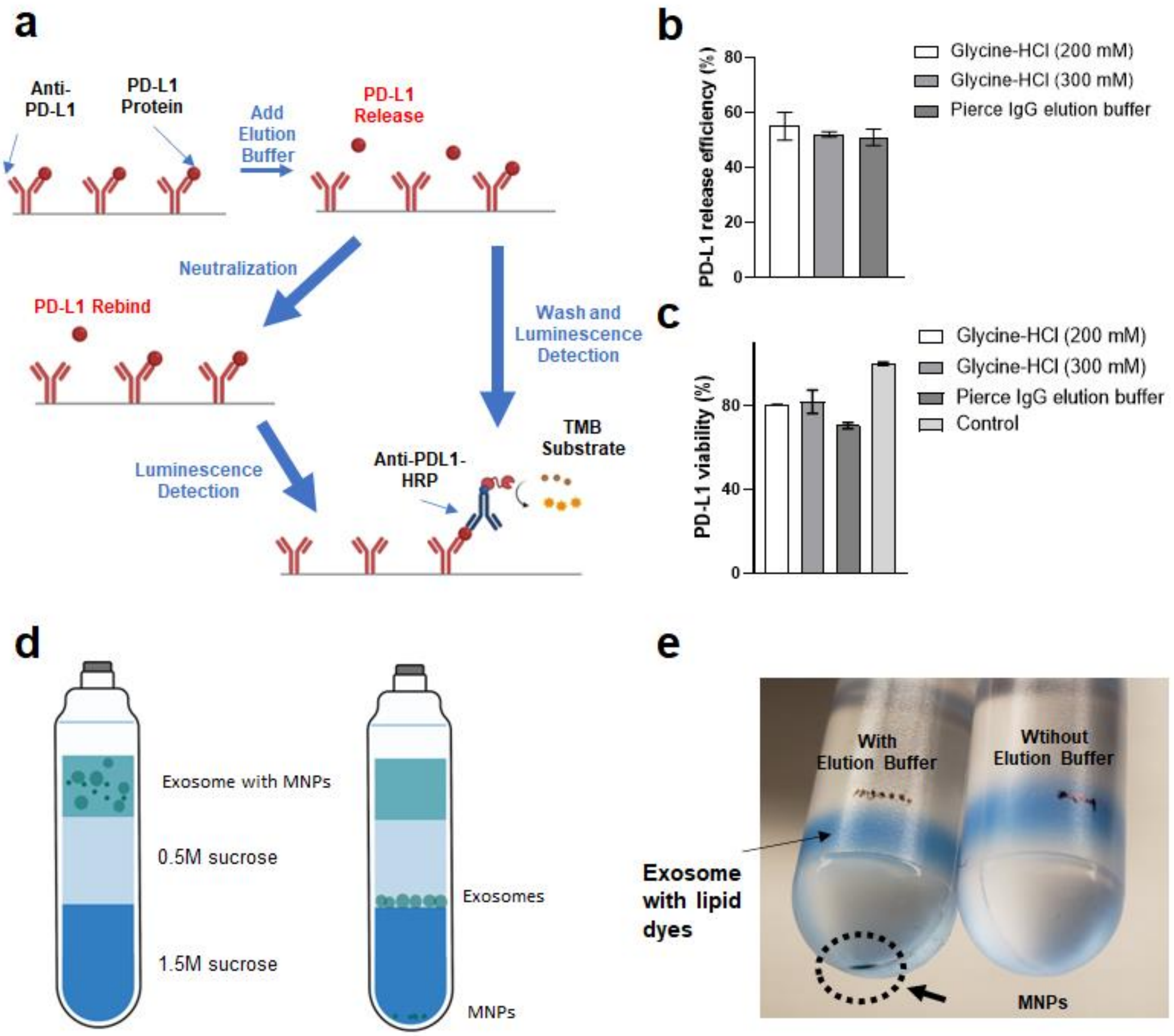

Supplementary Figure 9. **Evaluation of MNP release from sEVs. a**, Illustration of PD-L1 release assay and PD-L1 rebinding assay using ELISA. The assay portrays the efficacy of antibody release from sEVs and the integrity of PD-L1 on sEVs after treatment with elution buffer. **b**, Antibody release efficiency of PD-L1 from anti-PD-L1 after treatment with elution buffer. **c**, The integrity (viability) of PD-L1 was measured by rebinding released PD-L1 to anti-PD-L1 through the addition of neutralization buffer and compared with control (no elution buffer treatment). **d**, Schematic showing the separation of sEVs from released MNPs through sucrose gradient ultracentrifugation. **e**, Separation of sEVs from MNPs with sucrose gradient ultracentrifugation. SEVs were labeled with DiD lipid dye and collected in the interface of 0.5M and 1.5M sucrose layers. MNPs were pelleted at the bottom of the tube. The elution buffer is located above the 0.5M sucrose layer. Above data performed in duplicates, data represent mean ± s.d. Source data are provided as a source data file.

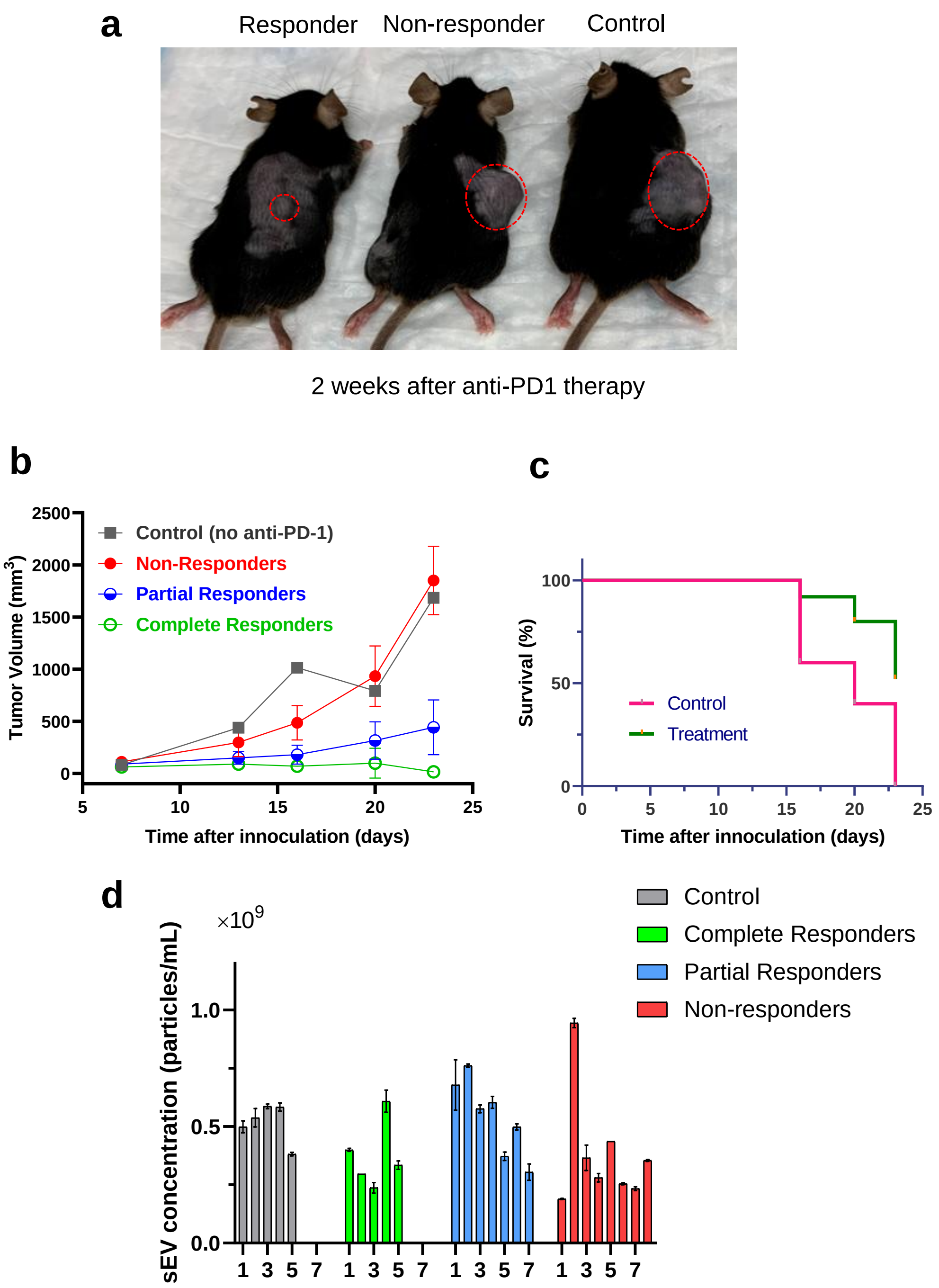

Supplementary Figure 10. **In vivo PD-1 immunotherapeutic model. a**, A representative image of mice from responder and non-responder to immunotherapy as well as control groups two weeks after anti-PD1 therapy. Data represents mean ± s.d. **b**, Tumor volume growth curve from all groups after inoculation of MC38 cancer cells. **c**, Survival curve of anti-PD-1 treated mice and untreated mice (control). **d**, sEV concentrations in plasma for mice in different groups. Source data are provided as a source data file.

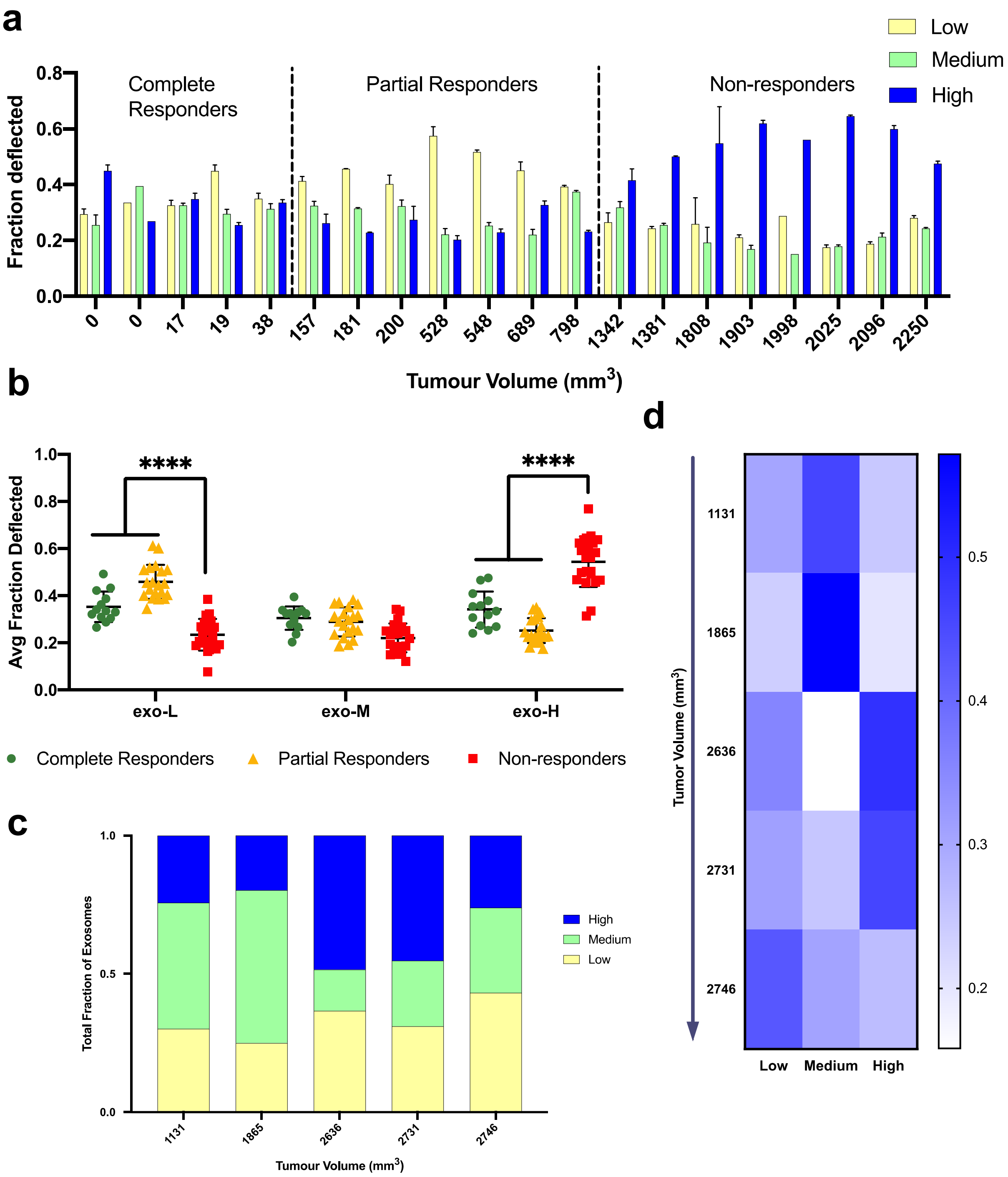

Supplementary Figure 11. exoPD-L1 profiling of PD-1 immunotherapeutic mouse model. **a**, exoPD-L1 profiling from the plasma of mice. The profiling pattern of responders, partial responders, and non-responders to immunotherapy are compared. **b**, Comparison of average exoPD-L1 distributions between complete, partial, and non-responders. **c**, exoPD-L1 profiling of the control mice (no PD-1 immunotherapy). **d**, Distribution of sEVs from control mice after sorting through NanoEPIC device. All data represent mean values, error bars represent s.d. ****P<0.0001, one-way ANOVA. Source data are provided as a source data file.

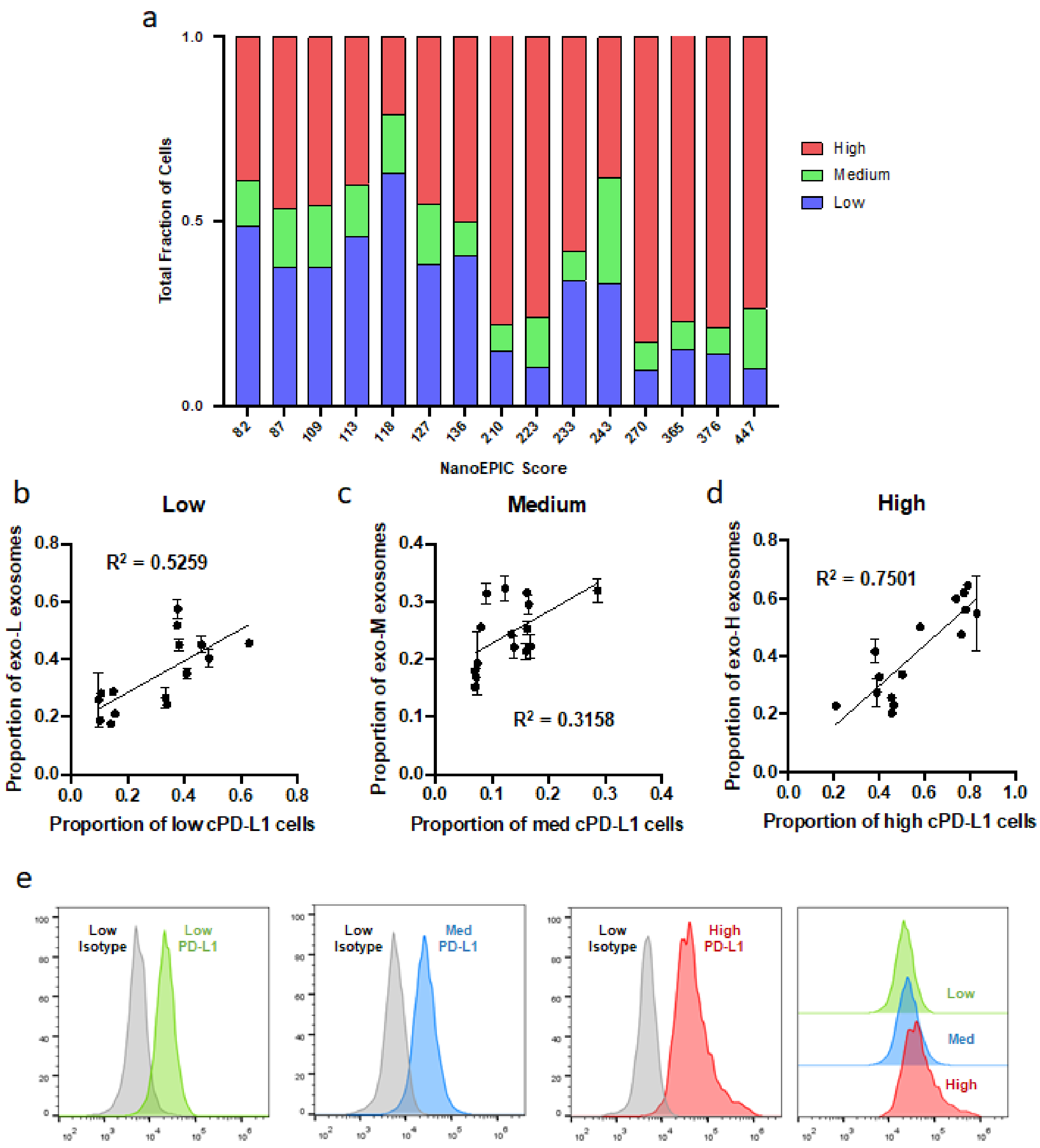

Supplementary Figure 12. **Assessment of the correlation between cPD-L1 and exoPD-L1. a**, cPD-L1 profiling of dissociated tumor cells from mice using PRISM chips. The cPD-L1 profiling pattern is compared with exoPD-L1 profiling through NanoEPIC score. SEVs and solid tumor cells were simultaneously sorted through the NanoEPIC and PRISM chip respectively. The proportion of exoPD-L1 and cPD-L1 in the low, medium, and high outlets were cross-examined (**b-d**). **b**, The proportion of exo-L compared with low cPD-L1. **c**, The proportion of exo-M compared with medium cPD-L1. **d**, The proportion of exo-H compared with high cPD-L1. **e**, Flow cytometric analysis of PD-L1 levels from MC38 cells sorted through the PRISM device for low, medium, and high cPD-L1. All data represent mean values, error bars represent s.d. Source data are provided as a source data file.

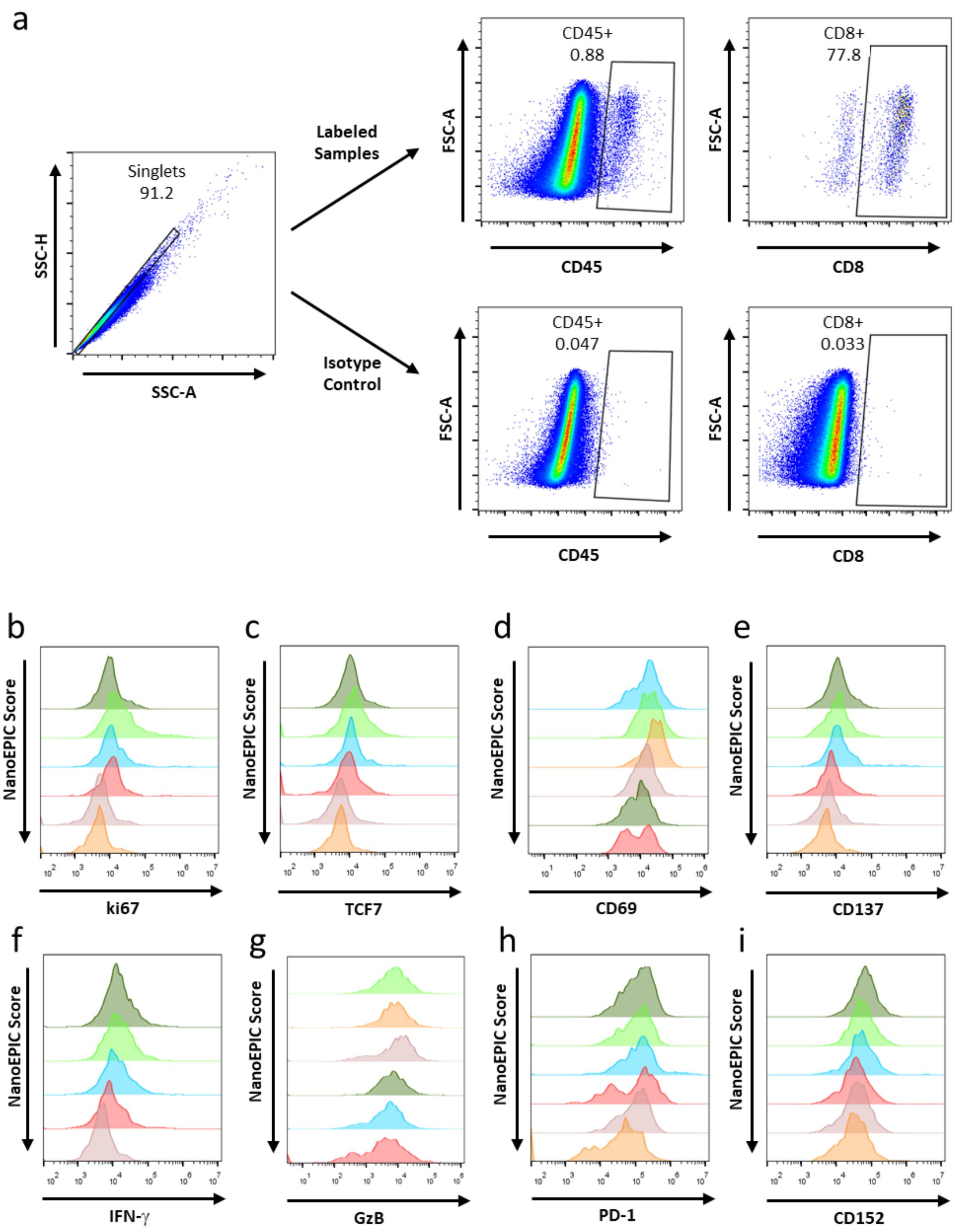

Supplementary Figure 13. Flow cytometric analysis of TILs from a murine immunotherapeutic model. a, Gating strategy for TIL analysis using flow cytometry. **b**, Flow cytometric analysis of proliferation markers ki67(**b**), T cell differentiation and activation markers (**c-e**), T cell cytotoxicity marker (**f, g**) and immune checkpoint related markers (**h, i**) shown in histograms arranged in ascending order of NanoEPIC score (top to bottom).

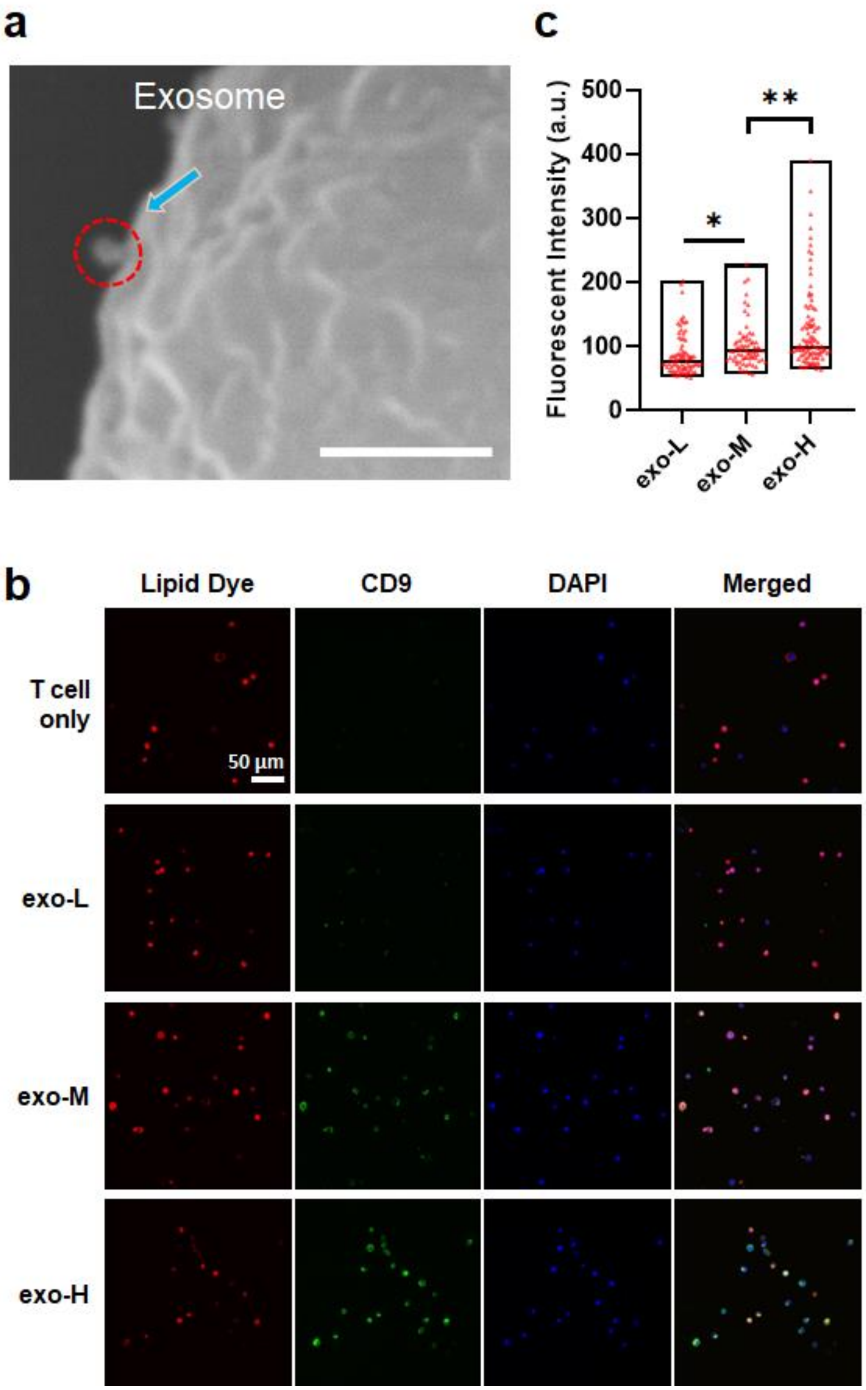

Supplementary Figure 14. **Imaging of sEVs bound to T cells. a**, A representative SEM image of an sEV (circled) bounded to T cells. Scale bar represents 1µm. **b**, Representative images of CD8+ T cells bound to different sEV subpopulations acquired by confocal microscope. T cells were labeled with lipid dyes (DiD) and nuclear stain (DAPI) while sEVs are labeled with anti-CD9-PE fluorescent antibodies. **c**, Measurement of CD9-PE fluorescent intensities for each T cell bound to sEVs from exo-L (n=83), exo-M (n=73), and exo-H (n=103). Box plots represent min, max, and median. *P<0.05, **P<0.01, unpaired t-test. Source data are provided as a source data file.

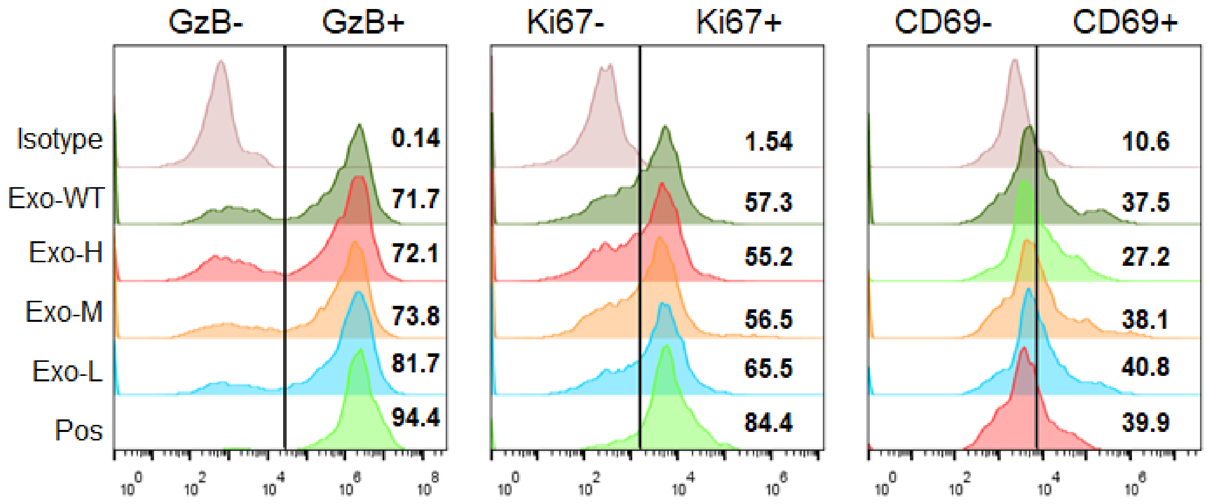

Supplementary Figure 15. Flow cytometric analysis of granzyme B, ki67, and CD69 from T cells after treatment with sEVs. Comparison of isotype control, T cells treated with wildtype sEVs (WT), exo-L, exo-M, or exo-H sEVs, and activated T cells with no sEV treatment (Pos). All sEVs were harvested from H1975 cells. Numbers represent the percentage of cells positive for the given marker.

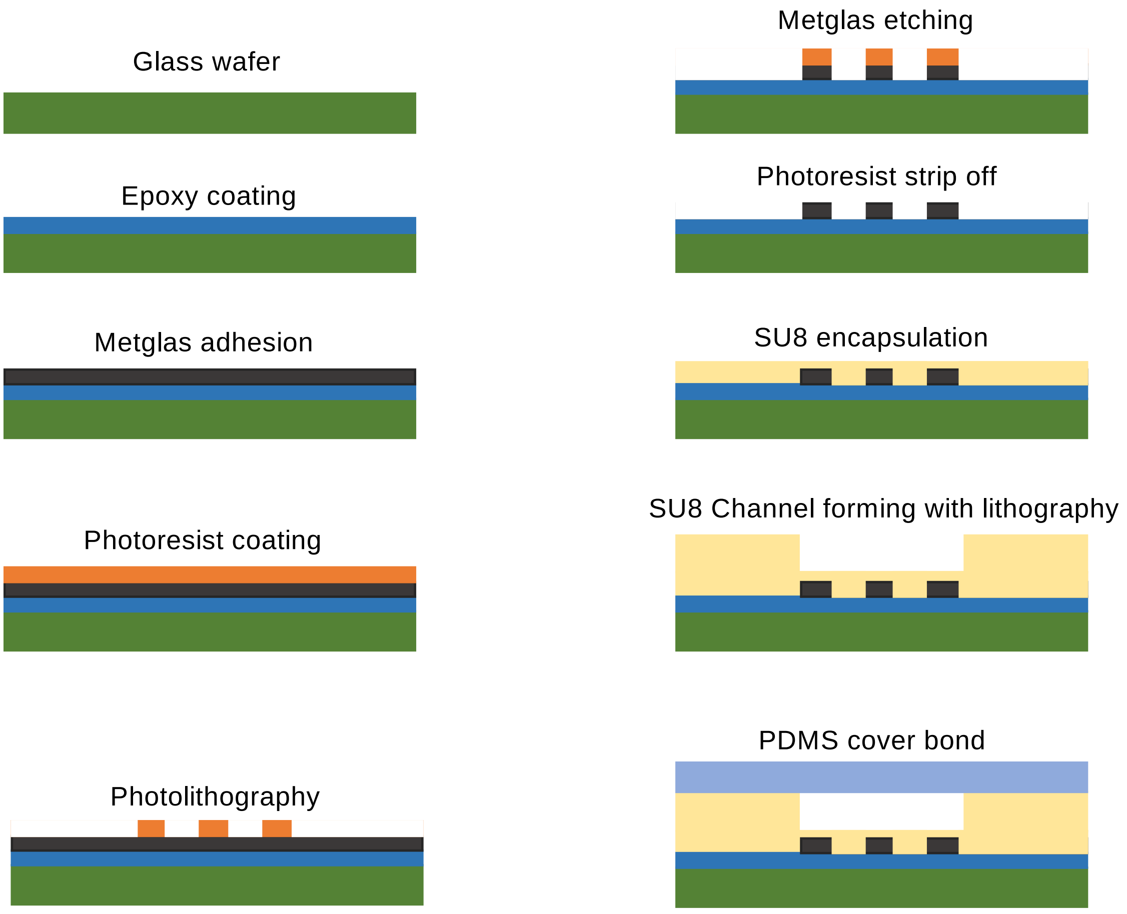

Supplementary Figure 16. Illustration of microfabrication steps for the NanoEPIC device.

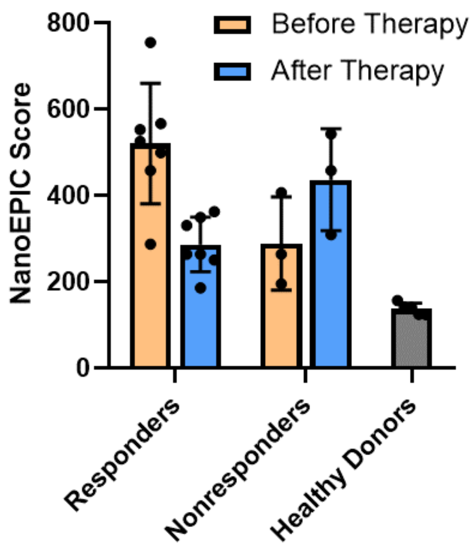

Supplementary Figure 17. NanoEPIC score of patients before and after anti-PD1/anti-PD-L1 immunotherapy. Responders generally show a decrease in NanoEPIC score while non-responders show increased in NanoEPIC score after immunotherapy.
