# Supplementary material for: A magneto-activated nanoscale cytometry platform for molecular profiling of small extracellular vesicles
Source: Nat Commun. 2023 Sep 11;14:5576. doi: 10.1038/s41467-023-41285-8 (PMC10495366; doi:10.1038/s41467-023-41285-8)
Supplement: Supplementary file 2 — Reporting Summary [file 41467_2023_41285_MOESM2_ESM.pdf]

nature portfolio

Corresponding author(s): Shana O. Kelley

Last updated by author(s): Jan 30, 2023

Reporting Summary

Nature Portfolio wishes to improve the reproducibility of the work that we publish. This form provides structure for consistency and transparency in reporting. For further information on Nature Portfolio policies, see our Editorial Policies and the Editorial Policy Checklist.

Statistics

For all statistical analyses, confirm that the following items are present in the figure legend, table legend, main text, or Methods section.

n/a	Confirmed	
☐	☒	The exact sample size (*n*) for each experimental group/condition, given as a discrete number and unit of measurement
☐	☒	A statement on whether measurements were taken from distinct samples or whether the same sample was measured repeatedly
☐	☒	The statistical test(s) used AND whether they are one- or two-sided<br>*Only common tests should be described solely by name; describe more complex techniques in the Methods section.*
☒	☐	A description of all covariates tested
☒	☐	A description of any assumptions or corrections, such as tests of normality and adjustment for multiple comparisons
☐	☒	A full description of the statistical parameters including central tendency (e.g. means) or other basic estimates (e.g. regression coefficient) AND variation (e.g. standard deviation) or associated estimates of uncertainty (e.g. confidence intervals)
☐	☒	For null hypothesis testing, the test statistic (e.g. *F*, *t*, *r*) with confidence intervals, effect sizes, degrees of freedom and *P* value noted<br>*Give P values as exact values whenever suitable.*
☒	☐	For Bayesian analysis, information on the choice of priors and Markov chain Monte Carlo settings
☐	☒	For hierarchical and complex designs, identification of the appropriate level for tests and full reporting of outcomes
☐	☒	Estimates of effect sizes (e.g. Cohen's *d*, Pearson's *r*), indicating how they were calculated

Our web collection on statistics for biologists contains articles on many of the points above.

Software and code

Policy information about availability of computer code

Data collection	Autocad: Chip design, CytExpert (v.2.4.0.28): Flow cytometry, Nanosight NTA NS300: nanoparticle tracking analysis, Hitachi HT7800: Transmission electron microscropy, Zeiss LSM 880: Confocal microscopy, Prisma E: scanning electron micrscopy, COMSOL Multiphysics: Simulation
Data analysis	FlowJo for flow cytometry (version 10.0.7), ImageJ (version 1.52a) for image analysis, Prism GraphPad (version 9.0.0) for data plot and statistical analysis, Microsoft Excel (version 16.69.1), MATLAB (R2020a)

For manuscripts utilizing custom algorithms or software that are central to the research but not yet described in published literature, software must be made available to editors and reviewers. We strongly encourage code deposition in a community repository (e.g. GitHub). See the Nature Portfolio guidelines for submitting code & software for further information.

Data

Policy information about availability of data

All manuscripts must include a data availability statement. This statement should provide the following information, where applicable:

- Accession codes, unique identifiers, or web links for publicly available datasets
- A description of any restrictions on data availability
- For clinical datasets or third party data, please ensure that the statement adheres to our policy

The main data supporting the results in this study are available within the paper and its Supplementary Information. Source data for the figures are provided with

this paper. Raw data and analyzed datasets for all chips, cells, mice, and human samples generated for this study are too large to be publicly shared, yet they are available from the corresponding author on request. Responses can be expected within four weeks.

Human research participants

Policy information about studies involving human research participants and Sex and Gender in Research.

Reporting on sex and gender	*Use the terms sex (biological attribute) and gender (shaped by social and cultural circumstances) carefully in order to avoid confusing both terms. Indicate if findings apply to only one sex or gender; describe whether sex and gender were considered in study design whether sex and/or gender was determined based on self-reporting or assigned and methods used. Provide in the source data disaggregated sex and gender data where this information has been collected, and consent has been obtained for sharing of individual-level data; provide overall numbers in this Reporting Summary. Please state if this information has not been collected. Report sex- and gender-based analyses where performed, justify reasons for lack of sex- and gender-based analysis.*
Population characteristics	*Describe the covariate-relevant population characteristics of the human research participants (e.g. age, genotypic information, past and current diagnosis and treatment categories). If you filled out the behavioural & social sciences study design questions and have nothing to add here, write "See above."*
Recruitment	*Describe how participants were recruited. Outline any potential self-selection bias or other biases that may be present and how these are likely to impact results.*
Ethics oversight	*Identify the organization(s) that approved the study protocol.*

Note that full information on the approval of the study protocol must also be provided in the manuscript.

Field-specific reporting

Please select the one below that is the best fit for your research. If you are not sure, read the appropriate sections before making your selection.

[x] Life sciences [ ] Behavioural & social sciences [ ] Ecological, evolutionary & environmental sciences

For a reference copy of the document with all sections, see nature.com/documents/nr-reporting-summary-flat.pdf

Life sciences study design

All studies must disclose on these points even when the disclosure is negative.

Sample size	No statistical methods were used to predetermine size. Sample sizes were selected based on previous experiences and benchmarked to most current studies in similar fields of work. For most cases, three biological triplicates or more were performed, unless otherwise noted. Due to limited sample availability, technical replicates were performed when dealing with mice plasma samples.
Data exclusions	No data was excluded
Replication	All experiments were performed with replication and shown in figures. All attempts for replication were reproducible.
Randomization	Animals were randomized into different treatment groups. All experimental were were randomly allocated into their experimental groups.
Blinding	The investigators were blinded during sample collection from mice and tumor-volume measurement and subsequent experimental analyses on samples, but were not blinded to the computation analysis as bias in the data is no longer an issue once collected. Investigator was blinded to group allocation during collection of samples.

Reporting for specific materials, systems and methods

We require information from authors about some types of materials, experimental systems and methods used in many studies. Here, indicate whether each material, system or method listed is relevant to your study. If you are not sure if a list item applies to your research, read the appropriate section before selecting a response.

Materials & experimental systems

n/a	Involved in the study
	[x] Antibodies
	[x] Eukaryotic cell lines
[x]	Palaeontology and archaeology
	[x] Animals and other organisms
	[x] Clinical data
[x]	Dual use research of concern

Methods

n/a	Involved in the study
[x]	ChIP-seq
	[x] Flow cytometry
[x]	MRI-based neuroimaging

Antibodies

Antibodies used

Dilutions and conditions were provided in methods section. See Supplementary Table 2
- anti-CD274, 13684, Cell Signaling Technology (https://www.cellsignal.com/products/primary-antibodies/pd-l1-e1l3n-xp-rabbit-mab/13684)
- anti-CD274, 14-5982-85, Thermofisher (https://www.thermofisher.com/antibody/product/CD274-PD-L1-B7-H1-Antibody-clone-MIH5-Monoclonal/14-5982-85)
- anti-CD63, ab271286, Abcam (https://www.abcam.com/products/primary-antibodies/cd63-antibody-kill150a-ab271286.html)
- anti-CD9, ab236630, Abcam (https://www.abcam.com/products/primary-antibodies/cd9-antibody-epr23105-121-ab236630.html)
- Anti-rabbit, HRP, 31460, Thermofisher (https://www.thermofisher.com/antibody/product/Goat-anti-Rabbit-IgG-H-L-Secondary-Antibody-Polyclonal/31460)
- Anti-mouse, HRP, 31430, Thermofisher (https://www.thermofisher.com/antibody/product/Goat-anti-Mouse-IgG-H-L-Secondary-Antibody-Polyclonal/31430)
- Anti-mouse IgG, Alexa Fluor 488, A28175, Thermofisher (https://www.thermofisher.com/antibody/product/Goat-anti-Mouse-IgG-H-L-Secondary-Antibody-Recombinant-Polyclonal/A28175)
- anti-CD274, APC, 17-5983-42, Thermofisher (https://www.thermofisher.com/antibody/product/CD274-PD-L1-B7-H1-Antibody-clone-MIH1-Monoclonal/17-5983-42)
- anti-Ki67, PE, 567719, BDbiosciences (https://www.bdbiosciences.com/en-us/products/reagents/flow-cytometry-reagents/research-reagents/single-color-antibodies-ruo/pe-mouse-anti-ki-67.567719)
- anti-Ki67, APC, 17-5699-42, Thermofisher (https://www.thermofisher.com/antibody/product/Ki-67-Antibody-clone-20Raj1-Monoclonal/17-5699-42)
- anti-TCF7, PE, 564217, BDbiosciences (https://www.bdbiosciences.com/en-us/products/reagents/flow-cytometry-reagents/research-reagents/single-color-antibodies-ruo/pe-mouse-anti-tcf-7-tcf-1.564217)
- anti-CD69, PE, 12-0691-83, Thermofisher (https://www.thermofisher.com/antibody/product/CD69-Antibody-clone-H1-2F3-Monoclonal/12-0691-83)
- anti-CD69, Super Bright 436, 62-0699-42, Thermofisher (https://www.thermofisher.com/antibody/product/CD69-Antibody-clone-FN50-Monoclonal/62-0699-42)
- anti-CD137, PE, 558976, BDbiosciences (https://www.bdbiosciences.com/en-us/products/reagents/flow-cytometry-reagents/research-reagents/single-color-antibodies-ruo/pe-rat-anti-mouse-cd137.558976)
- anti-IFN-g, PE, 554412, BDbiosciences (https://www.bdbiosciences.com/en-us/products/reagents/flow-cytometry-reagents/research-reagents/single-color-antibodies-ruo/pe-rat-anti-mouse-ifn.554412)
- anti-Granzyme B, PE, 12-8898-82, Thermofisher (https://www.thermofisher.com/antibody/product/Granzyme-B-Antibody-clone-NGZB-Monoclonal/12-8898-82)
- anti-Granzyme B (GB11), PE-Cyanine5.5, GRB18, Thermofisher (https://www.thermofisher.com/antibody/product/Granzyme-B-Antibody-clone-GB11-Monoclonal/GRB18)
- anti-PD-1, PE, 566831, BDbiosciences (https://www.bdbiosciences.com/en-us/products/reagents/flow-cytometry-reagents/research-reagents/single-color-antibodies-ruo/pe-rat-anti-mouse-cd279-pd-1.566831)
- anti-CD152, PE, 130-116-390, Miltenyi Biotec (https://www.miltenyibiotec.com/CA-en/products/cd152-antibody-anti-mouse-reafinity-rea984.html#conjugate=pe:size=150-ug-in-1-ml)
- anti-CD8a, APC, 561093, BDbiosciences (https://www.bdbiosciences.com/en-us/products/reagents/flow-cytometry-reagents/research-reagents/single-color-antibodies-ruo/apc-rat-anti-mouse-cd8a.561093)
- anti-CD8a, Super Bright 600, 63-0088-42, Thermofisher (https://www.thermofisher.com/antibody/product/CD8a-Antibody-clone-RPA-T8-Monoclonal/63-0088-42)
- anti-CD45, eFluor 450, 48-0451-82, Thermofisher (https://www.thermofisher.com/antibody/product/CD45-Antibody-clone-30-F11-Monoclonal/48-0451-82)

Validation

All antibodies used in this study were validated by the manufacturers, and validation data are available online at the manufacturers' websites. The antibodies were validated by flow cytometry and/or Western blotting and/or immunocytochemistry. Details about the used antibodies and the suppliers are provided in the previous section.

Eukaryotic cell lines

Policy information about cell lines and Sex and Gender in Research

Cell line source(s)

MC-38 was sourced from Kerafast. H1975, H460 were sourced from ATCC. PC9 was sourced from Sigma. Human Peripheral Blood Mononuclear Cells were sourced from Stem Cell Technologies.

Authentication

All cell lines had been authenticated by the original vendors. Cell lines from ATCC had been regularly tested and

	authenticated using morphology and PCR-based approaches to confirm cell identity.
Mycoplasma contamination	All cell lines were tested negative for mycoplasma contamination using Lonza's test kit, according to the manufacturer's protocol.
Commonly misidentified lines (See ICLAC register)	No misidentified cell lines were used.

Animals and other research organisms

Policy information about studies involving animals; ARRIVE guidelines recommended for reporting animal research, and Sex and Gender in Research

Laboratory animals	6–8 weeks-old female C57BL/6
Wild animals	The study did not involve wild animals.
Reporting on sex	Only female mice were selected to ensure no confounding variables with respect to gender would arise.
Field-collected samples	The study did not involve samples collected from the field.
Ethics oversight	The study protocol was approved by the University of Toronto Research Ethics Board.

Note that full information on the approval of the study protocol must also be provided in the manuscript.

Clinical data

Policy information about clinical studies

All manuscripts should comply with the ICMJEguidelines for publication of clinical research and a completedCONSORT checklist must be included with all submissions.

Clinical trial registration	Not applicable, as the study did not involve a clinical trial.
Study protocol	Not applicable.
Data collection	De-identified plasma samples of patients under anti-PD1/anti-PD-L1 immunotherapy were procured from PrecisionMed LLC. Plasma samples were processed with the NanoEPIC system.
Outcomes	Sorted sEVs were analyzed using Nanosight NS300. ExoPD-L1 profiling data were generated.

Flow Cytometry

Plots

Confirm that:

- [x] The axis labels state the marker and fluorochrome used (e.g. CD4-FITC).
- [x] The axis scales are clearly visible. Include numbers along axes only for bottom left plot of group (a 'group' is an analysis of identical markers).
- [x] All plots are contour plots with outliers or pseudocolor plots.
- [x] A numerical value for number of cells or percentage (with statistics) is provided.

Methodology

Sample preparation	Collected cells were incubated with the blocking buffer (1% BSA in PBS) for 15 min on ice. The cells were fixed with 4% PFA for 10 min on ice. For cell-surface protein analysis, the cells were incubated with fluorescently-labelled antibody for 30 min at room temperature. For intracellular protein analysis, the cells were permeabilized with 0.2% TX-100 for 10 min at room temperature after the fixation step and before incubation with the antibodies. Cells were washed three times with PBS between each step. Prior to analysis, cells were washed and re-suspended in PBS.
Instrument	CytoFlex S (Beckman Coulter)
Software	Data were acquired by using CytExpert v.2.4.0.28 and analyzed by using FlowJo_V10 software.
Cell population abundance	FACS was not performed. Gated fractions were all reported in associated figures and supplementary.
Gating strategy	Gating strategy was summarized in supplementary figure 13. Single-parameter histogram was used. All measurements were carried out relative to isotype controls.

- [x] Tick this box to confirm that a figure exemplifying the gating strategy is provided in the Supplementary Information.
